# Modelling Neanderthals’ dispersal routes from Caucasus towards east

**DOI:** 10.1371/journal.pone.0281978

**Published:** 2023-02-23

**Authors:** Elham Ghasidian, Anooshe Kafash, Martin Kehl, Masoud Yousefi, Saman Heydari-Guran

**Affiliations:** 1 Neanderthal Museum, Mettmann, Germany; 2 DiyarMehr Institute for Palaeolithic Research, Kermanshah, Iran; 3 Institute for Prehistoric Archaeology, University of Cologne, Koln, Germany; 4 Institute of Geography, University of Cologne, Cologne, Germany; 5 Department of Environmental Science, Faculty of Natural Resources, University of Tehran, Tehran, Iran; National Cheng Kung University, TAIWAN

## Abstract

The study of the cultural materials associated with the Neanderthal physical remains from the sites in the Caucasus, Central Asia and Siberian Altai and adjacent areas documents two distinct techno-complexes of Micoquian and Mousterian. These findings potentially outline two dispersal routes for the Neanderthals out of Europe. Using data on topography and Palaeoclimate, we generated computer-based least-cost-path modelling for the Neanderthal dispersal routes from Caucasus towards the east. In this regard, two dispersal routes have been identified: A northern route from Greater Caucasus associated with Micoquian techno-complex towards Siberian Altai and a southern route from Lesser Caucasus associated with Mousterian towards Siberian Altai via the Southern Caspian Corridor. Based on archaeological, bio- and physio-geographical data, our model hypothesises that during climatic deterioration phases (e.g. MIS 4) the connection between Greater and Lesser Caucasus was limited. This issue perhaps resulted in the separate development and spread of two cultural groups of Micoquian and Mousterian with an input from two different population sources of Neanderthal influxes: eastern and southern Europe refugia for these two northern and southern dispersal routes respectively. Of these two, we focus on the southern dispersal route, for it comprises a ‘*rapid dispersal route*’ towards east. The significant location of the Southern Caspian corridor between high mountains of Alborz and the Caspian Sea, provided a special biogeographical zone and a refugium. This exceptional physio-geographic condition brings forward the Southern Caspian corridor as a potential place of admixture of different hominin species including Neanderthals and *homo sapiens*.

## 1. Introduction

During the last few decades, great progress has been made in several domains, particularly palaeogenetics, which have revealed the complex ancestry of early Eurasians. This progress—including the identification of a “*ghost lineage”* of Eurasians in the Middle East—is providing important new biogeographical hypotheses [1]. Recent molecular and morphological research on the Neanderthal remains documents that this species appeared in Europe at least 400 ka [2–4] and ca. 150 ka in western Asia [5]. In their long-lasting period of occupation until ca. 30 ka, they colonized a vast territory that covered the entire European continent, the Levant and parts of Central Asia and Siberia [6–9].

Recent studies observed genetic variability among the Neanderthal groups and could potentially show high range of migration between them [10]. Mitochondrial DNA from Neanderthals found in Teshik-Tash in Uzbekistan and in the Altai region of southern Siberia shows that this species was of European origin [6]. Therefore, it can safely be concluded that they followed a long expansion route (at least 2000 km) eastward. The archaeological evidence and genetic sequence from Chagyrskaya and Denisova caves in the Siberian Altai confirm that Neanderthals migrated eastwards into Siberia. This process is understood not as a single dispersal event, but rather occurred repeatedly during the warm and temperate phases of the long time-span of MIS 5–3 (~130 to 28 ka) [8, 11–13].

In the Greater Caucasus, at Mezmaiskaya Cave, Middle Palaeolithic (MP) industry is known by foliate and Micoquian artefacts [14–16]. This industry resembles the one recorded from Chagyrskaya Cave. This cultural group, recovered from both Mezmaiskaya and Chagyrskaya caves and Central Trans-Urals sites [17], is considered to be sourced in Eastern Europe [8].

**Fig 1:**
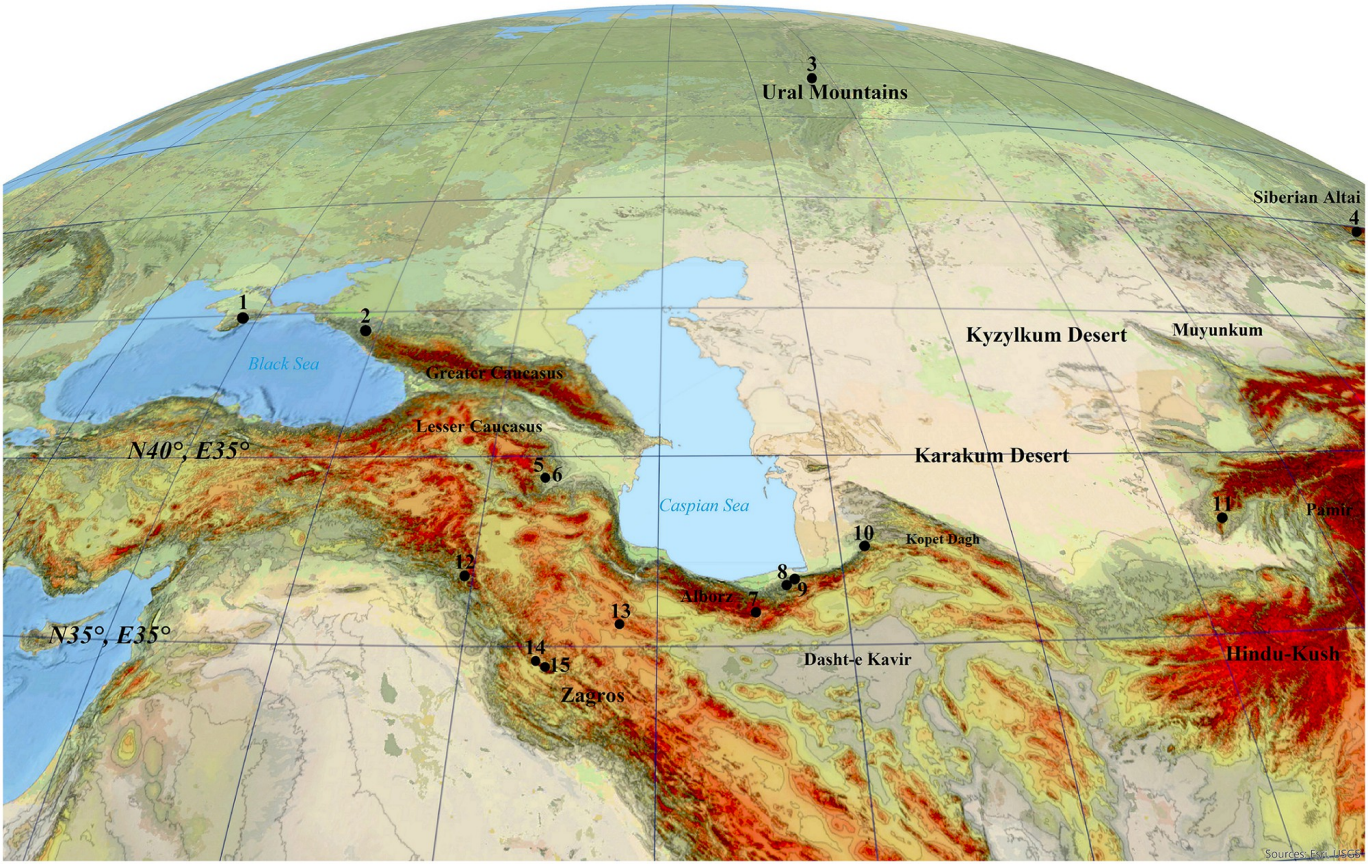
The study area and important Middle Palaeolithic sites mentioned in the text: 1. Crimean sites, 2. Mezmaiskaya, 3. Trans Ural sites, 4. Siberian Altai sites (Chagyrskaya, Denisova, Okladnikov), 5. Azokh, 6. Taglar, 7. Liben, 8. Shoupari, 9. Wezwar, 10. Keyaram, 11. Teshik-Tash, 12. Shanidar, 13. Qale Kord, 14. Bawa Yawan, 15. Bisetun.

Moving to the lower latitudes (ca. N 35–30°) (Fig 1), in the Lesser Caucasus, there is no sign of Micoquian. This issue gives rise to the hypothesis that Neanderthals split into different cultural groups in the Caucasus [14]. The Mousterian industry is well known in the Lesser Caucasus [18–20]. Research on the Azokh 1 Cave, in Lesser Caucasus, yielded Neanderthal remains associated with lithic artefacts ranging in age from Middle Pleistocene (MIS 9–8) to Late Pleistocene [20–24]. The Neanderthal physical remains and the associated MP lithic artefacts from this cave revealed a relatively contemporaneous age to the Zagros Mousterian from the Northern (i.e. Shanidar Cave: [25, 26]) and West-Central Zagros (i.e. Bawa Yawan Rockshelter: [27]) but older than the eastern-most Neanderthals’ territory in Central Asia [6].

At about 35 ka, a small and isolated group of Neanderthals appeared in Teshik-Tash Cave, in Uzbekistan; Central Asia. It is believed that this group of migrants was possibly assimilated, and their arrival did not result in long-lasting changes in the material culture of the local, possibly, Denisovan population [28]. The cave was excavated in 1930s and yielded Neanderthal remains named ‘Mousterian Child’ [29]. Brief analysis of the lithics at the time of excavation assigned them to the Mousterian cultural group [30]. The recent detailed lithic techno-typological analysis shows a hierarchical Levallois core reduction system, production of side scrapers and heavy-duty tools [31].

The current data proved the presence of Neanderthals in the whole Caucasus area located at the gateway of Europe and in Central Asia and Siberian Altai, as the eastern-most yet-known territory of this species (Fig 1). However, only few comparisons of material culture from the vast area between the Caucasus and Central Asia/Siberian Altai have been conducted [8]. Data on how, when and which route the Neanderthals took towards East remain unclear. Sites in the regions between this western and eastern points are still low in number [32, 33]. These shortcomings together with little information on the climatic and topographic characteristics of this vast area during the Last Glacial constraints the study of the Neanderthals’ dispersals towards east.

The Caucasus area has a rather complicated picture in this time. Climatic deterioration phases of Late Pleistocene perhaps limited the Neanderthals’ contact between Greater and Lesser Caucasus. Palaeolithic cultural varieties recognised in this area divided the Caucasus into at least two biogeographical and cultural groups of northern (Micoquian) and southern Caucasus (Mousterian) which fits with the geographical division of the mountains into Greater and Lesser Caucasus. The high species diversity of fauna and endemism in the Caucasus demonstrates it as a biodiversity hot spot [34] connecting two geographical realms of the western Palearctic (i.e. Euro-Siberian) and the Irano-Turanian [35]. The Lesser Caucasus is considered as part of the Eastern Mediterranean–Black Sea–Caspian-Corridor [36] for Pleistocene hominin dispersals towards Central Asia. The presence of the western Palearctic glacial forest refugium of Hyrcania located between Alborz Mountains and the Caspian Sea [34] makes the larger region a hot spot for the study of hominin diversity and dispersal. This area is geoecologically connected to the Lesser Caucasus via Talysh Mountains, forming a corridor and potentially the shortest dispersal route (2000 km), that the Neanderthal populations took towards Central Asia. Recent studies of changes in the biogeography of other organisms than humans, and their constituent populations during Late Pleistocene [34], bring forward a model for the Neanderthals’ adaptation and dispersals. During MIS 4, the climatic conditions in the Southern Caspian Sea corridor (SCC) was relatively humid and mild [37, 38] in comparison to the neighbouring areas, including interior parts of the Iranian Plateau. Therefore, the SCC could have acted as a refugium for Late Pleistocene hominins including Neanderthals.

In this regard, we present a robust biogeographical model using Least-Cost-Path (LCP) analysis to characterize Neanderthal population dispersals with distinct material cultures from Caucasus (Greater and Lesser) towards east (Siberian Altai). Our focus is the time frame of MIS 4 (71–57 ka), the period that has contained extreme cold and dry climatic condition [39].

## 2. Methods

### 2.1. Corridor mapping

LCP analysis is the most commonly used method to reconstruct dispersal corridors of ancient hominins [40]. In this study we applied LCP analysis to model dispersal route of Neanderthals between archaeologically significant locations; Mezmaiskaya and Azokh caves in the Caucasus with Micoquian and Mousterian cultural materials respectively as two starting points and Chagyrskaya and Denisova caves in the Altai Mountains, Russia to cover both Eastern Micoquian and Mousterian cultural groups [8, 14, 20, 21, 29, 41]. We consider the Altai mountains sites (namely Chagyrskaya and Denisova caves) as one ending point as they offer similar physiogeographic condition and less than 100 km distance [8] and contain two cultural entities of Micoquian and Mousterian.

In our LPC model, we assume the Caspian Sea, as the world’s largest closed sea located between Caucasus and Central Asia, was a natural barrier. Accordingly, every dispersal route towards east must be passed either from the vast plains located in the Russian Plain in the north or the narrow land of SCC in the south. Caspian Sea level experienced high fluctuations during different periods. The present water level is at– 27 m asl. While transgressions occurred during interglacial (Khazarian highstand at -10 m asl during MIS 5) and some pleniglacial periods (Early and Late Khvalynian highstands at 50 and 0 m asl during Late MIS 3 and MIS 2), during the first cold stage of the Last Glacial (Atelian lowstand during MIS 4) the water level was even lower than present, possibly as low as -140 m asl [42]. In addition, the southern Caspian Lowlands is an area of tectonic subsidence, causing accumulation of thick sedimentary deposits. Transgression of the Sea and tectonic subsidence probably removed possible traces of hominin settlements (see e.g. [43]) or buried them under thick younger sediments. As Sea level transgression during Late MIS 3 reached a high stand of 50 m asl and possibly removed all hominin settlement traces, we considered areas with less than 50 m elevation as not suitable areas for tracing hominin dispersal in our model. However, during Caspian Sea regressions the coastal lowlands were significantly larger, providing ample space for dispersal. In addition, our methodology is based on the energetic costs and subsistence benefits. Another potential barrier for dispersal is the Karakum Desert located on the southern dispersal route (Fig 1). Today the Karakum desert is an area with frequent dust storms [44]. During the Pleistocene, aridity was even higher and aeolian activity stronger, which can be indirectly deduced when considering the increased dust accumulation rates forming loess deposits in the Iranian loess plateau [45, 46] and along the northern foothill zone of the Alborz Mountains [47–51] or in Tajikistan [52]. Recent research hypothesises that the Karakum desert could be one of the potential dust sources of north-eastern Iranian loess [53, 54] indicating very cold and dry and perhaps impassable conditions during glacial periods. In the lack of MP evidence, we thus assume that, due to the increased aridity, the Karakum desert could not be passed by Neanderthals and could be considered as high costly feature to the expansion route. Therefore, we redirected dispersal routes on the mountain piedmonts. This feature has an impact on both speed and direction of the dispersals. Our interest is focused on the finer topographic details of the mountain piedmonts that follows the theory of ‘*high contrast topography*’ applied already for the MP sites of the Zagros [55]. The MP sites are mostly located at the edge of corridors close to the high topographical contrast of mountainous regions overlooking the plains. These corridor systems canalized movement patterns of games from different ecological patches [55].

LCP was performed using QGIS 3.16 [56]. Since slope is known as a critical factor affecting travel rates and route arrangement while hiking, jogging, or running along a trail [57], we considered areas with higher slope as areas with higher movement cost for the Neanderthals. Slope is an important factor in the amount of energy costs on the uneven aspects of the route including cliff faces [58]. Thus, to build a cost raster layer, we calculated slope of each cell within the study area using the *terrain* function in the raster package [59]. The *terrain* function calculates slope as a function of elevation [59]. The elevation layer was obtained from the Shuttle Radar Topography Mission (SRTM) elevation model [60].

### 2.2. Estimating climate stability

Areas which experienced little climatic fluctuations acted as refugia for different taxa including mammal species [61]. Thus, we speculated that areas which remained stable during the past climatic changes were used as refugia by hominin populations. To identify climatically stable regions across the study area, we calculated temperature change velocity from the Last Interglacial (LIG; ~120,000–140,000 years BP) towards the Last Glacial Maximum using the following R packages; raster [59], gdistance [62], SDMTools [63], matrixStats [64]. Climatic data for the LIG and the LGM as the last most important interglacial and glacial stages were downloaded from PaleoClim and CHELSA respectively [65–67]. PaleoClim.org is a source of free, high-resolution Paleoclimate data for use in biological modelling and GIS. CHELSA (Climatologies at High resolution for the Earth’s Land Surface Areas) is a very high resolution (30 arc sec, ~1km) global downscaled climate data set currently hosted by the Swiss Federal Institute for Forest, Snow and Landscape Research WSL.

We also mapped precipitation and temperature of MIS 4 for the study area in QGIS 3.16 [56] using paleoclimate data obtained from the Oscillayers dataset [68].

## 3. Physiogeography of the SCC

The SCC area is bounded by the northern slopes of the highly rugged and east-west oriented Alborz Mountain ranges that separate the inner lowland deserts of the Iranian Plateau in the south from the Caspian Sea in the north. Despite being narrow, this area includes diverse geographical elements; from sand dunes close to the Sea shore in the north to the high Alborz Mountains in the south. A wide *elevational gradient range*, diverse topography and strong environmental heterogeneity offered a flora and faunal diversity. In its eastern-most part, Alborz Mountains join the Kopet-Dagh-Khorassan Mountains, while from north-west and west it connects to the Lesser Caucasus and Zagros respectively. Biogeographical studies show that the Alborz Mountains is the host of numerous local endemics acting as a strong east-west biogeographical link [69].

The SCC is partly covered by the Caspian Hyrcanian forest. This forest covers around 55,000 square kilometres. Its western extension reaches to the Lesser Caucasus. Dating back to between 25 to 50 ma, Caspian Hyrcanian mixed forest has been refugium for numerous fauna and flora species persisting during glacial intervals. The Caspian Hyrcanian forest is ecologically important because it contains many already extinct or endangered species elsewhere as well as the highest local endemic flora diversity in the Iranian Plateau [69, 70]. This great diversity among flora of the Hyrcanian region is partly due to its geographical isolation [71, 72]. Its unique biogeographical nature provides long-term preservation of different flora and faunal species and ecosystems represented across the Hyrcanian region. It provided a suitable place for the evolution of flora over time and acted as a large reservoir for it [71, 72]. Phylogeographical studies of flora and faunal species shows the persistence of several species during glacial periods in the SCC introducing it as ‘*refugium of Hyrcania’* [34, 73].

**Fig 2:**
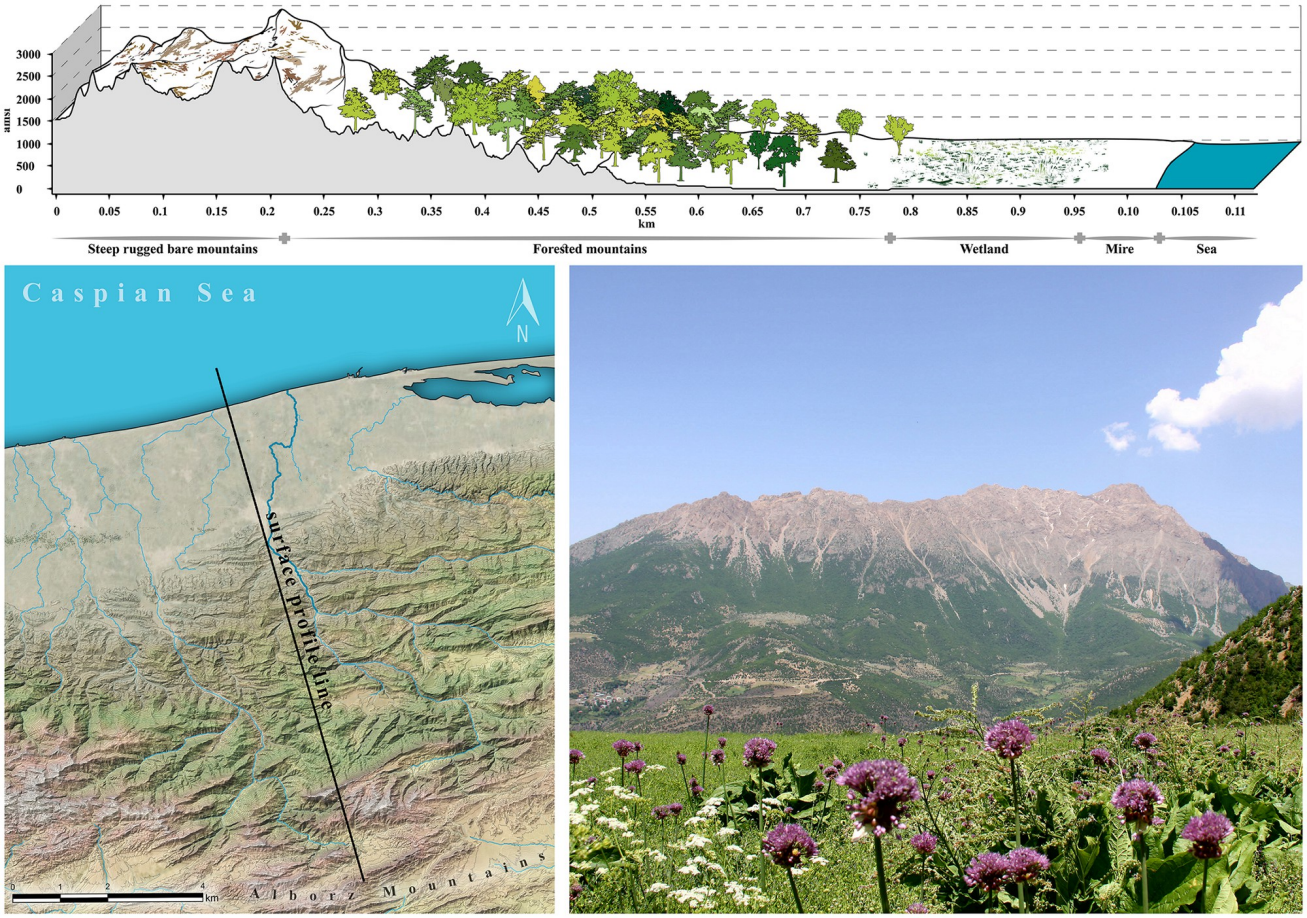
Heterogeneous environment of Southern Caspian Corridor. Figure on top is the reconstruction of the surface profile line depicted in picture low left. Contrast of mountain and plain is depicted on photo low right (photo credit A. Bavand Savadkouhi).

The heterogeneous environment (mountains, forests, wetland, mire, sand dunes) provides diverse niche space hypothesising SCC as a reservoir for detecting hominins genetic variation, biodiversity, biogeographical development and evolution. Based on patterns observed from the other parts of the Iranian Plateau (mainly Zagros), we observed that in the altitude above ca. 1800 meters, the hominin settlements decreased [55]. Therefore, we considered areas higher than 50 m (far from water level fluctuations) up until 1800 m asl as SCC for hominin dispersal and settlement (Fig 2).

### 3.1. Environment and climate: Past and present

Palaeolithic settlements in SCC were influenced by different elements including climatic conditions and sea level fluctuations.

Our main data on the paleoclimate of the Iranian Plateau during MIS 5 comes from speleothem growth and δ18O records of Qale Kord [74] and Pir Ghar [75] caves located in the northeast edge of the Zagros Mountains and in the east of the Iranian Plateau, respectively. Further information is provided by the palynological study of a sediment core from Lake Urmia [76]. Increased arboreal vs. non-arboreal pollen shows that MIS 5e was a comparatively humid and temperate phase. This issue is also evident from the formation of forest soils in loess-palaeosol sequences (LPS) of the Southern Caspian Lowlands [77]. After the last interglacial, at least two more humid periods probably correlating with interstadials of MIS 5 and interrupted by dry and cool stadials have occurred [74–78] The Paleoclimate of MIS 4, as main focus of our paper, is poorly described. However, it is considered as having similar condition as MIS 2 [58].

Generally, MIS 4 is associated with cold and probably dry climatic conditions in the high altitudes of the Caucasus and in many places of the Iranian Plateau including the Alborz highlands. Palynological data from Caucasus shows that during the stadials, continental cold and arid climatic conditions, caused by the influence of glaciers, covered a huge area [79]. As a result, specific flora types, including periglacial meadow-steppes, pine, alder, and birch have appeared [14].

**Fig 3:**
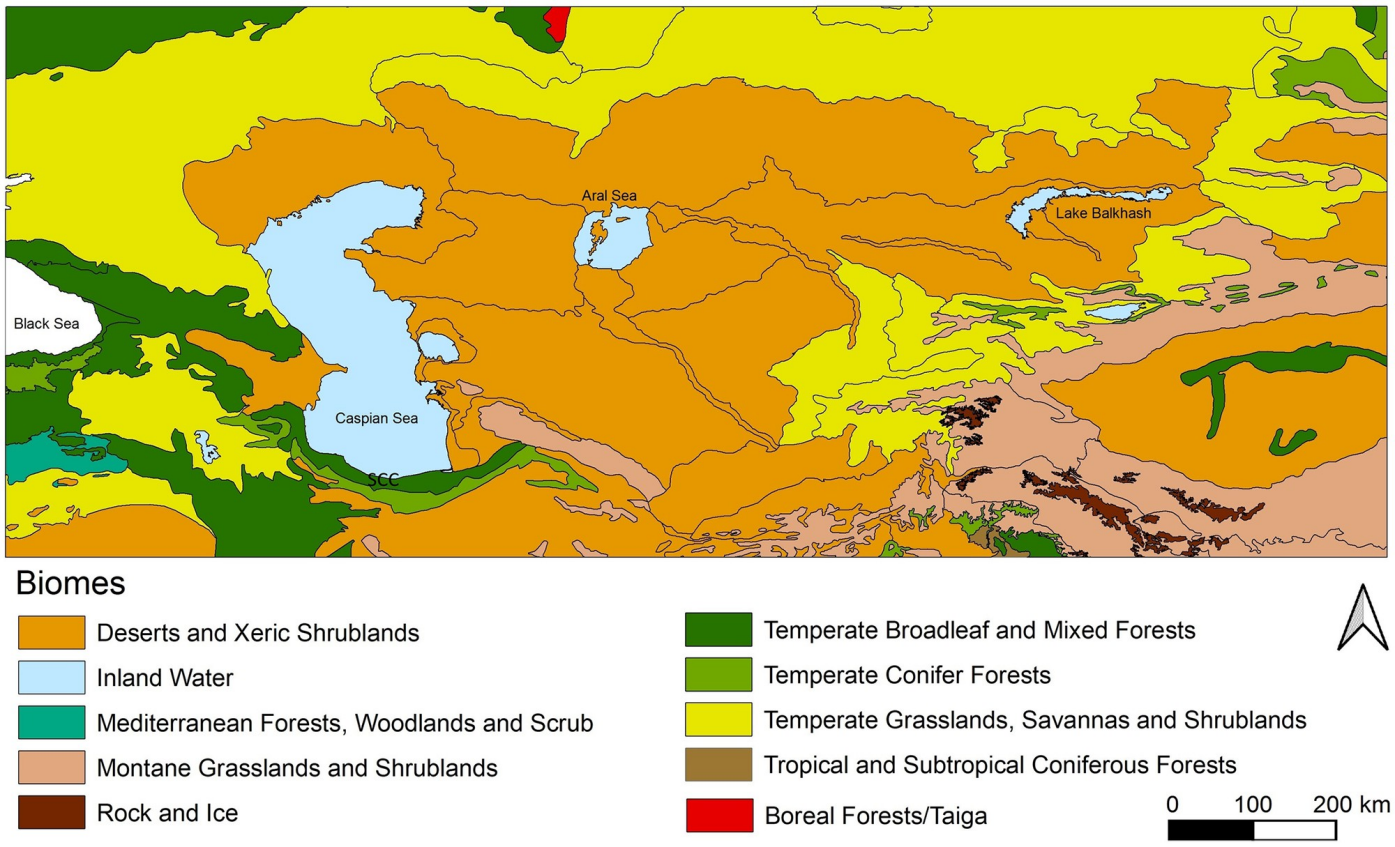
Distribution of terrestrial biomes in the study area. The Southern Caspian Corridor (SCC) is covered by Temperate Broadleaf and Mixed Forests. The biomes data is based on the World Wildlife Fund (WWF) Terrestrial Ecoregions [119].

Palaeoclimatic records from Lake Urmia in Iran documents a cold and arid phase during MIS 4 [76, 80]. MIS 4 was, most probably, the driest time in the SCC. The loess records of the eastern SCC show that after MIS 5, dust accumulation rates strongly increased whereas intensity of soil formation decreased [50, 77]. It is very likely that the forest retreated towards the western parts of the SCC and steppe vegetation, spread from the coast towards the montane areas. After MIS 4, dust accumulation rates reduced and intensities of soil formation increased again resulting in several weakly developed syngenetic soils subdividing the loess deposits. Climatic conditions remained comparatively dry until the early MIS 2, when a more strongly developed palaeosol could form after a change to more humid conditions [50, 77]. With the LGM, aridity increased again causing accumulation of the uppermost loess layers in which the modern soil could form under moister climatic conditions similar to the present day. The loess-palaeosol sequences thus document changes in humidity in the SCC during the last Glacial to Holocene. In addition, Caspian Sea level changes may have governed local climatic conditions increasing aridity during low stands and increasing humidity during high stands thus affecting the timing of soil forming periods in the SCC during the last glacial. The special physiogeographic condition of the SCC makes it different from the neighbouring areas as observed in modern time (Fig 3).

**Fig 4:**
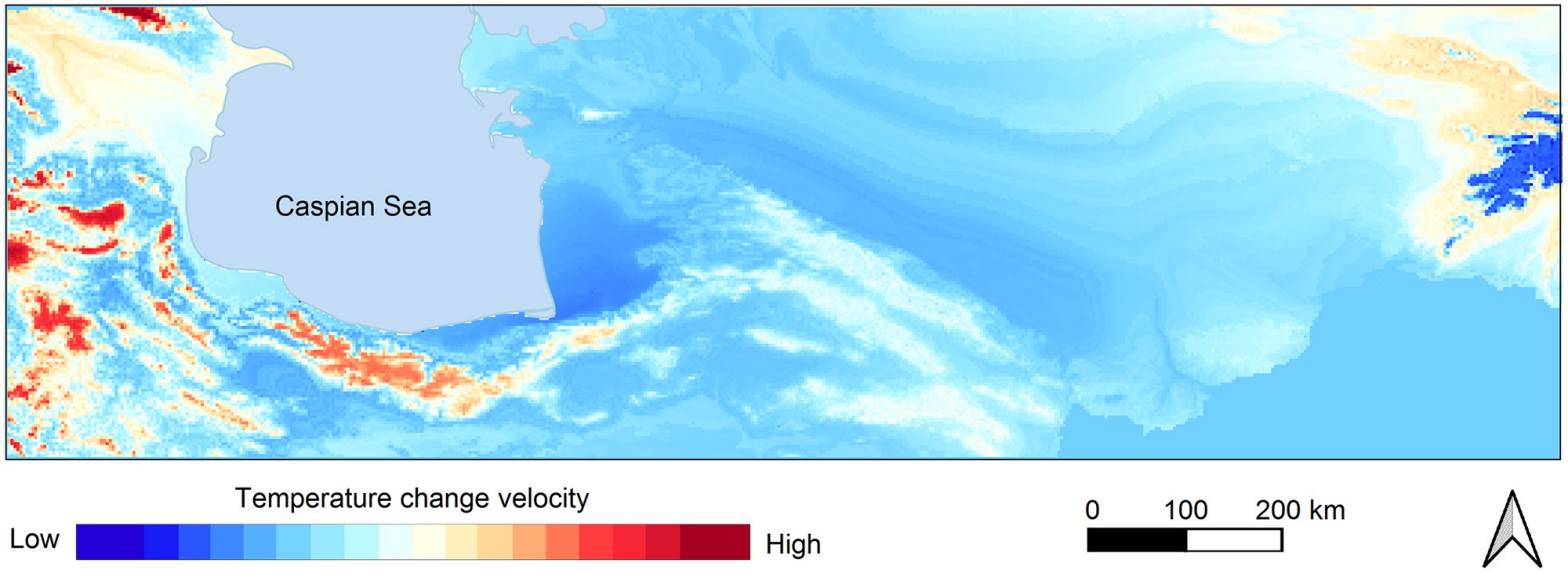
Present day temperature change velocity in Southern Caspian Sea Corridor and neighbouring areas.

**Fig 5:**
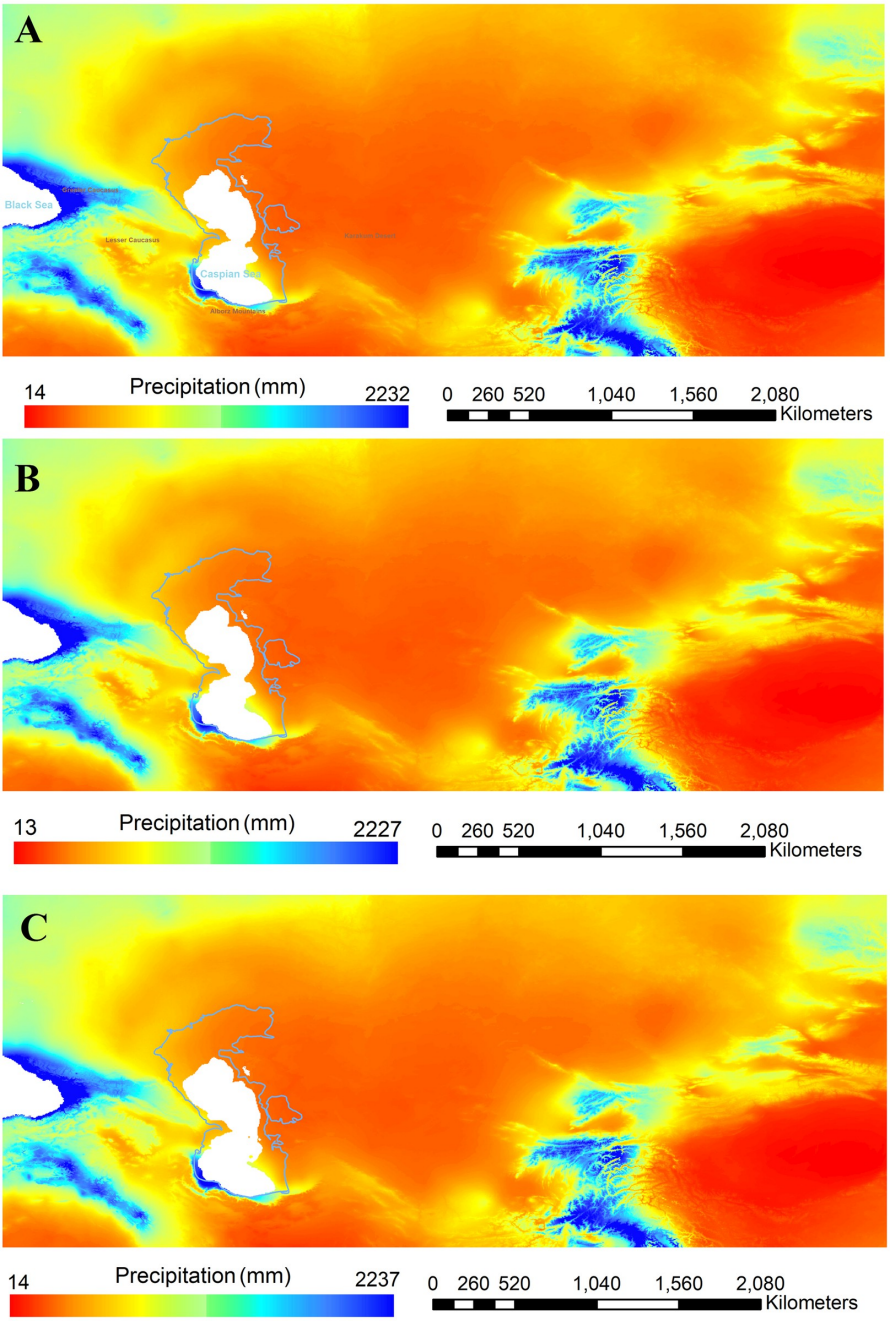
Reconstruction of spatial variations of annual precipitation during MIS 4. A: 50 ka, B: 60 ka, C: 70 ka (maps created based on Oscillayers dataset^68^).

**Fig 6:**
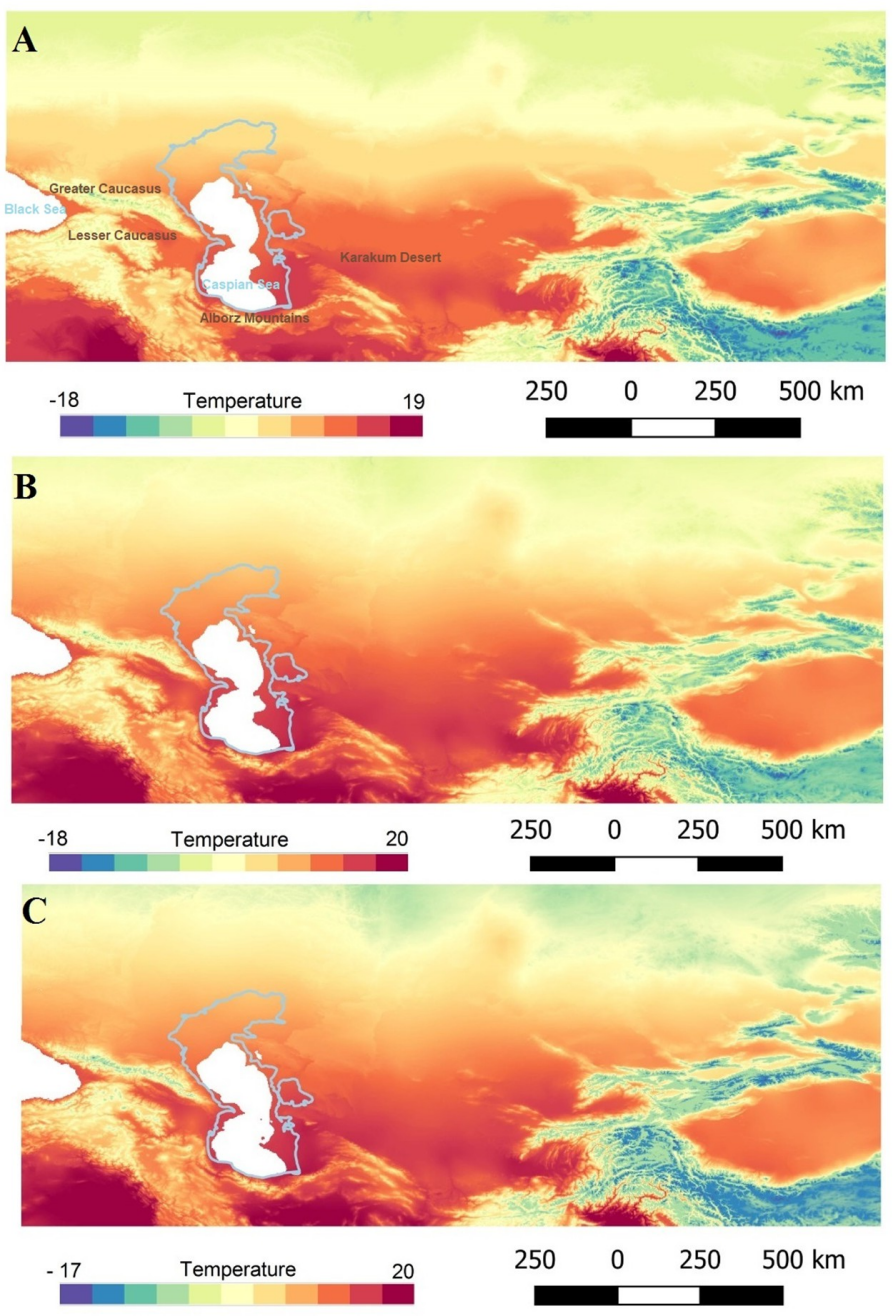
Reconstruction of spatial variations of annual temperature during MIS 4. A: 50 ka, B: 60 ka, C: 70 ka (maps created based on Oscillayers dataset^68^).

The present-day climate of the SCC is influenced by the Alborz Mountains in the south, the Caspian Sea in the north, the Caucasus Mountains in the west and deserts and steppe landforms in the east and northeast (Fig 4). Mean annual precipitation rates range from around 1800 mm in the westernmost to 300 mm in the easternmost part of the corridor and are thus considerably higher than on the southern slopes of the Alborz Mountains where 250 mm are recorded. This difference demonstrates the special climate of the SCC. The Caucasus and Alborz Mountain ranges promote rainfall in the SCC, by blocking the Westerly winds bringing moisture from the Mediterranean, Black Sea and Caspian Sea Annual temperature and precipitation data for MIS 4 [68] gives prominence to this area (Figs 5 and 6), showing that the area benefitted from milder climatic conditions and rich water and food resources.

### 3.2. Caspian Sea level fluctuations

The Caspian Sea is one of the barriers on the dispersal routes between west and east [81]. The shores around the world’s largest closed sea were highly affected by the regressions and transgressions during glacial and interglacial cycles [42]. However, Caspian Sea level changes follow a different pattern than the global sea level changes. Since Caspian is a closed basin, it is highly influenced by climate as well as hydrographic factors including the drainage system of rivers, mainly the Volga [82].

**Fig 7:**
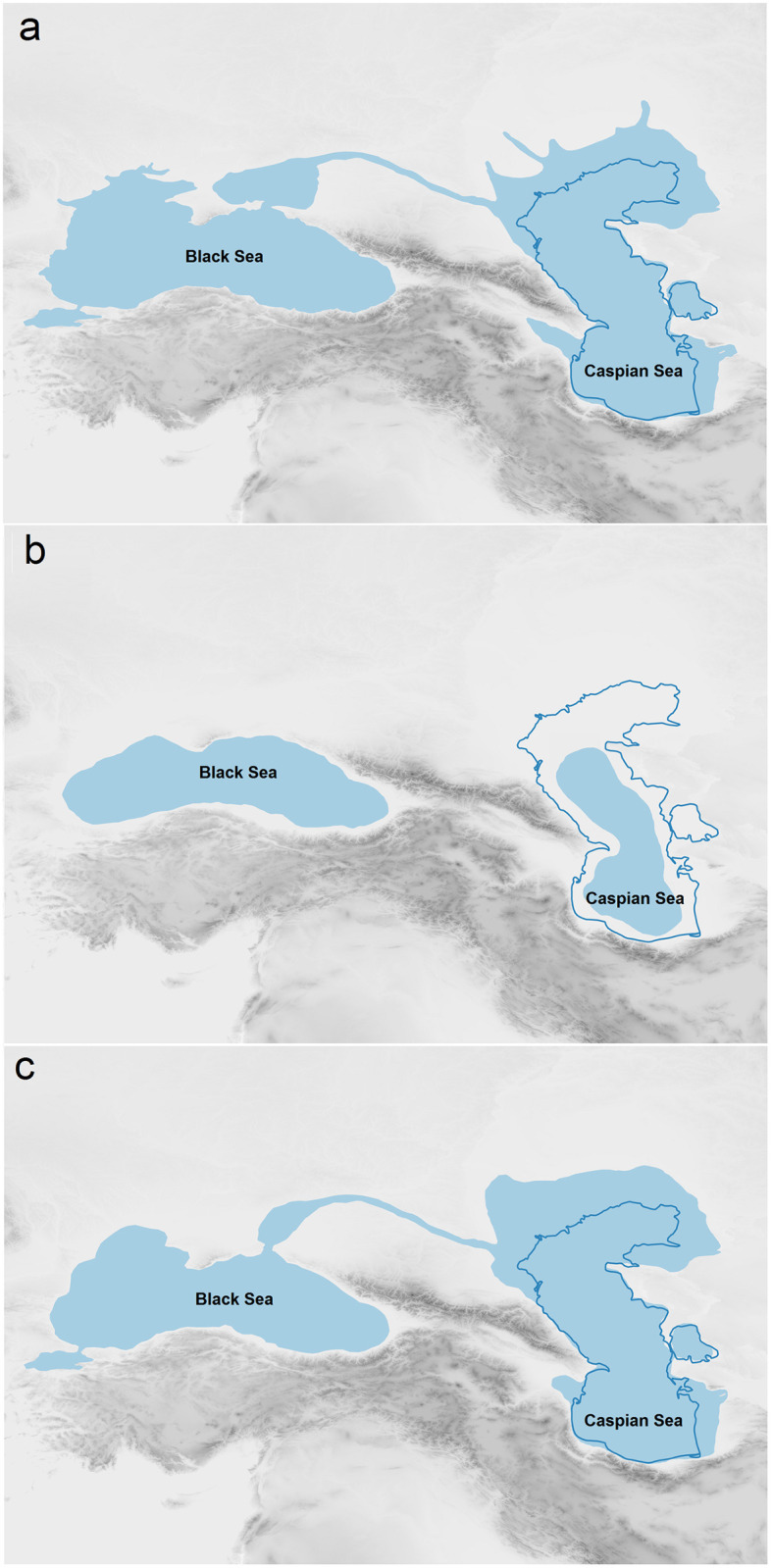
Reconstruction of Caspian Sea level at late Pleistocene. A: MIS 5e (Late Khazarian transgression), B: MIS 4 (Atelian regression), C: MIS 3 (Khvalynian transgression).

Caspian Sea level changes depended on the hydro-climatological processes caused by water evaporation and precipitation during different glacial and interglacial cycles [81, 82] (Fig 7). During warm interglacial periods, melting of the ice sheets caused increasing river discharge. Even the Central Asian rivers like Amu Darya and Syr Darya flowed into the South Caspian Basin [83, 84]. In the Last Interglacial stage of MIS 5e, the Caspian Sea level experienced a large transgression named Late Khazarian (roughly dated between 114 and 75 ka), up until the MIS 4 regression. The Late Khazarian transgression corresponds to a sea level high stand of -10 m [85].

During MIS 4 the Caspian Sea level was at the minimum level. This stage, which is locally named Atelian lasted to around 48 ka [42, 83, 86]. The maximum low stand during the Atelian is estimated at -120 to -140 m exposing vast areas around the Caspian Sea [87–93]. Recent research on the northern coasts of the Caspian Sea documents mammalian remains including mammoth, horse and reindeer in the Atelian deposits. These species indicate the presence of tundra-steppe and cold-arid continental climate [83] at the north of the Caspian Sea. Towards the end of the Atelian stage, the climate became warmer changing the flora types (birch, pine and spruce trees). Elm, oak and linden re-appeared, and grasses and herbaceous vegetation expanded resulted into expansion of steppe and forest-steppe environments [94–97].

After the Atelian regression, the Caspian Sea high stand of the Early Khvalynian transgression occurred [83]. It was probably caused by increased surface run-off of the Volga River and resulted in overflow from the Caspian towards the Black Sea basin [83]. This transgression occurred between ca. 35–21 ka which partly correlates with the global interstadial warming of the later phases of MIS 3 [98]. At the beginning of MIS 3 the Caspian Sea level started to rise and reached up to 50 m asl towards MIS 2 [99]. During MIS 2 at LGM, namely Khvalynian, the sea level decreased dramatically. This situation came to an end during the warm phases of the Bølling and Allerød interstadial which caused rise of the Caspian Sea level [83]. Pollen records of the Caspian Sea basin [82] and alluvial plains [100] indicate a dry and cold period during the LGM that changed gradually to the moist and warm conditions during the Holocene. Data on the exact timing and nature of climatic oscillations after MIS 5 and before the Last Glacial Maximum (LGM) on the Iranian Plateau, generally, and SCC, in particular, is thus still limited.

## 4. Results

Based on the transgression and regression pattern of the Caspian Sea, we suggest dispersal routes and timings of the Neanderthals’ dispersals via SCC and during MIS 4 (Atelian regression).

MP sites with Neanderthal physical remains are scarce in the study region. This is due to the small scale of excavations. However, few sites at the extremities of the study region yielded physical remains associated with material culture including Mezmaiskaya and Azokh caves in Greater and Lesser Caucasus respectively, Teshik-Tash in Central Asia, Denisova, Okladnikov and Chagyrskaya caves in the Siberian Altai. In western Iranian Plateau in the Zagros Mountains, Shanidar Cave and Bawa Yawan Rockshelter and in central western Iran Qale Kord Cave yielded Neanderthal remains associated with the MP Mousterian techno-complex [101].

In addition, we plotted the sites with MP material culture confirming hominin occupation during MIS 5–3. In the lack of absolute dating, a rough chronology has been applied for the sites. Our goal, however, was not to create a specific ‘date’ for the sites, but instead to place them within a glacial or interglacial cycles of Late Pleistocene.

### 4.1. Towards a model for the Neanderthal dispersals to east

**Fig 8:**
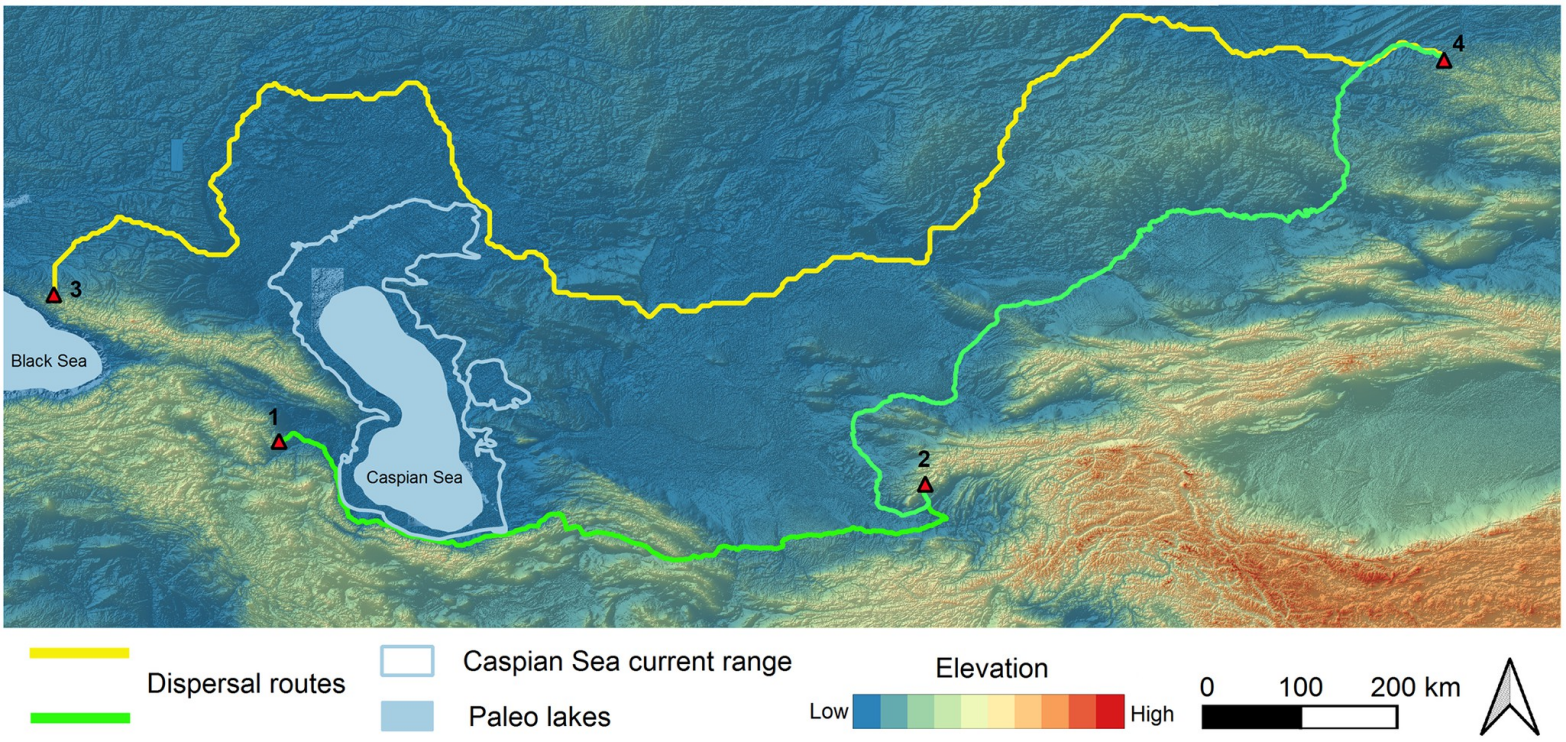
Least-cost-path from Caucasus towards east. 1: Azokh, 2. Teshik-Tash, 3. Mezmaiskaya, 4. Chagyrskaya. The yellow line indicates the Northern Caspian dispersal route and the green line indicates the Southern Caspian dispersal route.

For conducting route determination for two possible dispersal routes from Caucasus towards Siberian Altai, we chose two starting points in the Caucasus which yielded Neanderthal fossil remains associated with MP lithic artefacts (Fig 8). Mezmaiskaya Cave in the Greater Caucasus was occupied by the Neanderthals during MIS 5–3 and yielded Eastern Micoquian cultural group [14, 15, 102]. However, the absolute lack of Micoquian in the Lesser Caucasus compelled us to choose another starting point in this region representing Mousterian as both entities are present in the Siberian Altai sites at the end point. The Azokh Cave in Lesser Caucasus yielded Mousterian assemblage associated with the Neanderthal fossils [19, 22–24]. Our model also determines and anticipates the barriers such as Caspian Sea and Karakum desert which restrict and slow down the dispersals across them. The Caspian Sea and its coast was considered as the main barrier, since during different climatic fluctuations, the regression and transgression of the Caspian Sea level influences hominin dispersals and the preservation of archaeological evidence.

### 4.2. Northern dispersal route: Greater Caucasus to the Siberian Altai

The northern dispersal route has its start point on Mezmaiskaya Cave on Greater Caucasus and the end point on the Chagyrskaya Cave in Siberian Altai, since the two sites share similar cultural materials of Micoquian. The northern dispersal route assumed to be used in the time period of 70 to 55 ka [8]. Generally, the Neanderthals of Micoquian cultural group were considered to be specialized in horse and bison hunting and were adapted to steppe and piedmont environments [8, 103, 104].

The MP Micoquian settlements are known in the Central Trans-Ural Mountains between the starting and ending points along the northern dispersal route [17]. The route passes the northern coasts of the Caspian Sea as the largest barrier. In the lack of any high and impassable mountains, the slope factor was easily applied in our model. The presence of Volga delta on the north Caspian forced the computer to track a route away from its fluctuation, for it is a dissected delta and is/was highly influenced by sea level fluctuation (Fig 8). Its low gradient is gentler than any other major delta system and because of the vast north Caspian plain, any small sea level fluctuations caused its horizontal dislocations [84].

Genetic and archaeological data [8, 12], hypothesise that this route along the Eurasian steppe belt during cold and arid climatic condition was the direct route from eastern Europe towards the Siberian Altai. In this view, the Neanderthals have crossed the Pontic-Caspian Region, penetrating the southern latitudes into the Greater Caucasus but not any further south to the Lesser Caucasus. The Pontic-Caspian Region is seen as a favourable settlement area populated often by different hominins including Neanderthals during Late Pleistocene [83].

Despite of the presence of Micoquian at Mezmaiskaya, this cultural group is totally absent in the Lesser Caucasus. We hypothesise that the severe climatic condition during stadial period (MIS 4) has limited contact between the settlements in Greater and Lesser Caucasus. The Lesser Caucasus has generally lower altitude and a more arid climate than the Greater Caucasus. This issue limited proceeding ice sheets to 700 m asl during glacial periods [105]. It has been suggested that the Likhi Mountain Range prohibited humid air masses from the Black Sea to the inner parts of the Lesser Caucasus area and caused arid condition for the northern foothills of the Lesser Caucasus [106]. It also separated refugium of Colchi from the eastern lowlands of Lesser Caucasus area including Kura Basin and further south Hyrcania Refugium [34].

It has been proposed that the reason behind the difference between MP techno-complexes on northern and southern sides of the Caucasus, namely Eastern Micoquian with foliates of the north and the Mousterian with different kinds of scrapers from the south, is the impassable mountains which acted as a “*cultural boundary*” [107]. This boundary was even stronger during the harsh climatic stage of MIS 4 [108]. The dissimilarity of MP assemblages, and in the later period, the similarity between the Upper Palaeolithic (UP) assemblages across the Caucasus, highlights the role of Caucasus as a “*biogeographical and social barrier”* for the Late Pleistocene populations [107]. *Homo sapiens* could better spread across Caucasus during the interstadial stages and climatic amelioration [108].

### 4.3. Southern dispersal route: Lesser Caucasus to the Siberian Altai via SCC

McBurney in his mission to Iran in 1960s, proposed that any hominin movement from west might be expected to pass SCC *en route* to the Central Asia [109]. The Upper Jurassic limestone in this region contains caves, some of which appear to preserve substantial depths of deposit [43]. This region also provided rich stone raw materials like chert and flint nodules embedded in limestone deposits [110].

Here, we evaluate the “*rapid dispersal route*” [58] by generating LCP from the starting point at Azokh Cave on the southern piedmonts of the Lesser Caucasus to the east. The southern dispersal route passes the narrow area between the Caspian Sea and the Alborz Mountains through the Hyrcanian biogeographical refugium namely SCC on the northern piedmonts of Alborz, Kopet-Dagh along northern foothills of Hindu-Kush and Pamir Mountains to the Hissor Mountain range towards Tian Shan and terminates to the Siberian Altai.

**Fig 9:**
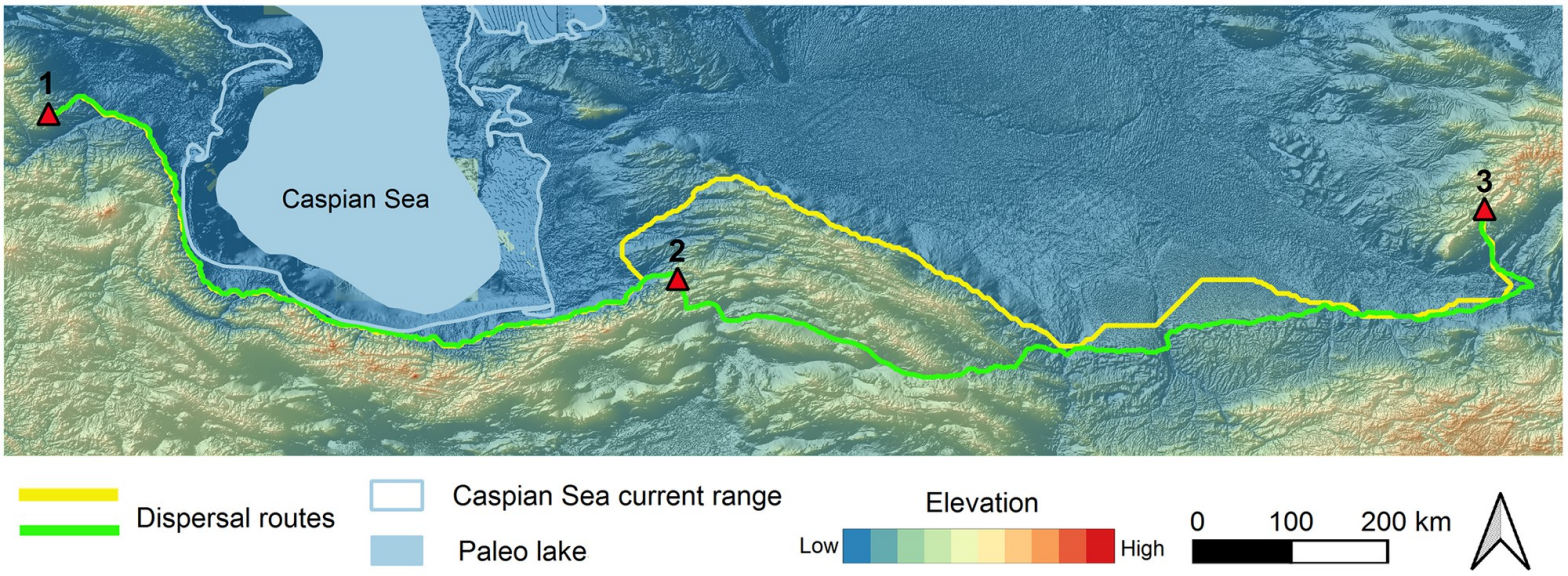
Southern Caspian Sea dispersal route. 1. Azokh, 2. Keyaram, 3. Teshik-Tash.

**Fig 10:**
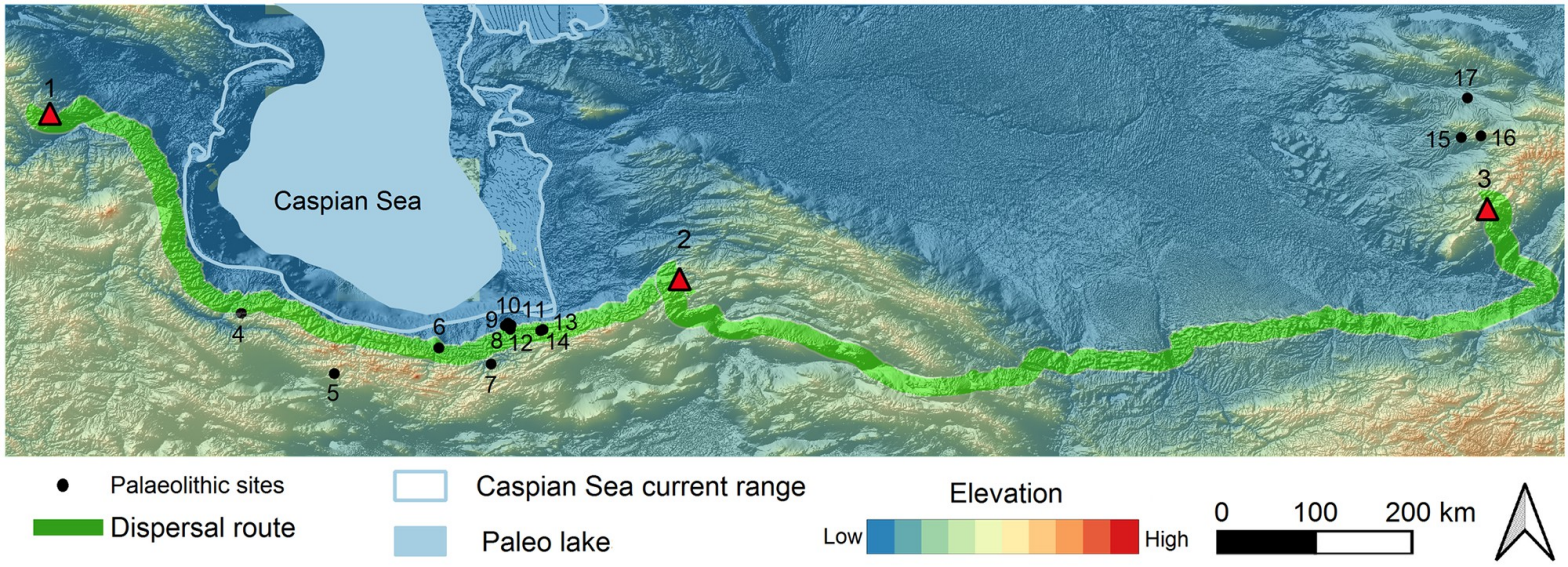
The southern dispersal route with 30 km buffer zone as highly potential hominin settlements (1. Azokh and Taglar, 2. Keyaram, 3. Teshik-Tash, 4. Darband, 5. Eskouldar, 6. Garmroud, 7. Liben, 8. Komishani, 9. Kolet, 10. Rostamkola, 11. Shoupari, 12. Khanesar, 13. Wezwar, 14. Kiyasar, 15. Anghilak, 16. Aman Kutan, 17. Khodjamazgil.

For evaluating the rapid dispersal route towards east, a number of values have been considered to find the best route including climate, topography and actual and potential shelter sites along the suggested route. In addition to simulation using these variables, we examine the “*high contrast topography*” model employing the environmental and geographic conditions, once suggested for the early UP sites in the Southern Zagros Mountains of Iran [55]. This model assists to recognise the dispersal route in the areas yet empty from the archaeological finds including southern Turkmenistan and northern Afghanistan. Our focus, therefore, is on the mountain piedmonts overlooking plains monitoring games that would have accelerated or hampered hominin dispersals towards east. This reflects that the initial expansion from Caucasus required SCC transversal route of SCC which was predominantly a moist and temperate area consisting numerous caves and rock shelters with terrestrial and aquatic food resources. However, towards the end of this corridor, the moisture and temperature conditions are reduced dramatically. Here we predict the dispersal routes along the northern piedmonts of Kopet-Dagh and Hindu-Kush Mountains (Figs 9 and 10).

The glaciation intervals of MIS 4 led to global decreases in sea-levels and increasing aridity caused the desert condition in some regions [58]. During this time, SCCver in arid Central Asia is developed as a belt along the southern and south-eastern margins of the Karakum, Kyzylkum, Muyunkum and Gurbantunggut deserts hypothesising these deserts acted as barriers for hominin dispersal during these time spans. High dust accumulation rates in the Iranian Loess Plateau during the Last Pleniglacial [45, 46, 49] reflect very dry climate conditions at the eastern end of the SCC. Because of increased aridity during the Pleniglacial, we consider the Karakum desert as a barrier (Fig 8). Thus, the computer program generated the route in two main parts: the first starts from Azokh to Teshik-Tash Cave in Central Asia and the second from Teshik-Tash to the Siberian Altai. For the second part, two routes have been suggested. The one passes the northern piedmonts of Kopet-Dagh and the other one runs through the mountain valleys and plains south of Kopet-Dagh (Fig 9). We predict that both piedmonts and mountain valleys have potentially hosted hominin (including Neanderthals) settlements. On the northern foothills of Hindu-Kush Mountains, these two routes join together towards Siberian Altai.

In general, the SCC with ca. 800 km length has a central role in the southern dispersal route. Geologically, the SCC is an area of tectonic subsidence, whereas Alborz Mountains experienced tectonic uplift [111, 112]. This caused elevational diversity between at least -27 m in the Lowlands and generally 3000 m asl in the mountains, resulting in an unusual biogeographical status of the SCC in Western Asia with Mediterranean climate, dense vegetation, permanent rivers, and coastal and marine food resources throughout the year. Coupled with numerous caves and rockshelters, the SCC represented a diverse geographical region in favour of Late Pleistocene populations. The Alborz Mountains in the south has limited the interactions between this area and the inner parts of the Iranian Plateau.

Where the northern Caspian Sea was highly affected by the sea level fluctuations, the southern coast remained relatively less affected by regression and transgression of the sea during glacial and interglacial cycles [42]. This natural circumstance becomes highly important in terms of the possible interaction between different hominin species arriving from west and east ending up in the meeting point of SCC.

## 5. Discussion

Northern and Southern Caspian routes presented in the dispersal model of the Neanderthals towards east here, are hypothetical and formed based on archaeological and physiological data in hand. Regarding Neanderthals’ dispersals, this model generates several hypotheses for future scope of research in a relatively unknown areas of Iran and the Central Asia. From two routes suggested by LCP modelling, the southern route shows that the narrow area of SCC, provides the closest and fastest and more optimised route connecting Caucasus to Central Asia and eventually the Siberian Altai.

Preliminary archaeological data indicates that this area probably acted as dual role of biogeographical corridor of expansion and habitat and witnessed a series of human evolutionary events that occurred during Late Pleistocene [113].

### 5.1. Neanderthals’ refugium

The biogeographical studies in SCC document this area as a biodiversity hot spot for flora and faunal endemism. Despite its limited size, this area fosters a high number of local endemics as well as acting as biogeographical connections between west towards east [69].

During glacial periods Siberia could not serve as refugium, therefore, the areas further south including the Levant and Zagros have been suggested as potential refugia [114]. Due to the lack of information, the SCC refugium is always ignored. Based on the reconstruction of annual precipitation and annual temperature during MIS 4 (Figs 5 and 6), we suggest that this area, in addition to the southern Europe and south-western Asia, could serve as a refugium for Neanderthals, as it was for other species (see e.g. [34, 69, 71–73]. The location of SCC connected to the Caucasus as an immediate area at the gate of Europe fits with the model of southern refugia [114]. SCC could act as a refugium for the hominin species coming from both west and east sides, namely Neanderthals from west and other hominin species (including possibly Denisovans from the east i.e. Siberian Altai). For proving that hominin fossils are crucial.

### 5.2. Archaeological evidence

**Fig 11:**
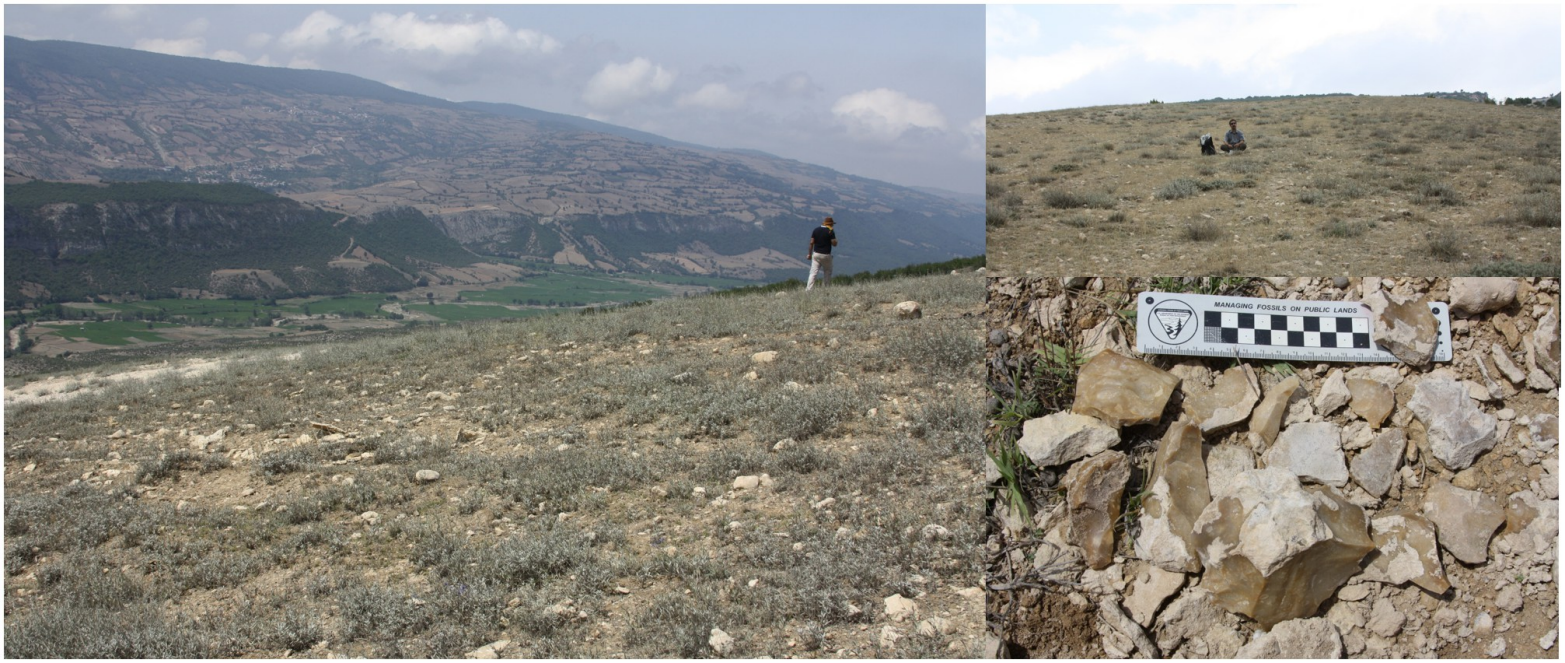
Wezwar open air site in eastern SCC (photos by E. Ghasidian).

Recent survey in SCC especially in the eastern part of it, led to the discovery of several Palaeolithic sites, including shelter and open-air sites [113]. Fig 10 shows the distribution of the Late Pleistocene sites in this corridor and two east and west extremities. The 30 km buffer along the LCP here, indicates the highly potential place for hominin settlements including Neanderthals. So far, several settlements have been recognised in this area. Among them, are Liben Cave at the centre and Shoupari Cave and the large MP open-air site of Wezwar located at eastern part of SCC [113]. Wezwar is formed on a four-square km geological formation including eroded limestone beds associated with fine grained chert nodules [113]. On its surface, thousands of artefacts are scattered including typical MP artefacts associated with Levallois flakes and blades resembling the contemporaneous sites in both Lesser Caucasus and in the Zagros (Fig 11).

Our dispersal model shows two separated LCPs confirmed by two cultural material groups of Micoquian and Mousterian on Greater and Lesser Caucasus respectively. The absolute lack of Micoquian at Lesser Caucasus points to the lack of communication between two parts of the Greater and Lesser Caucasus during MIS 4. The bearers of Micoquian techno-complex in Eastern Europe took a long journey over 4000 km to the complex of sites in Siberian Altai (e.g. Chagyrskaya and Okladnikov) [8] penetrating northern parts of the Caucasus, as was observed in Mezmaiskaya. The lack of Micoquian in the Lesser Caucasus is interesting. We interpret this as the population replacement during glacial period (MIS 4) from different sources (e.g. *southern Refugia*; [114]). The re-colonization of Lesser Caucasus at this period was possible through the migration from refugia of southern parts including SCC back to Caucasus. Therefore, SCC could act as a population reservoir during climatic deterioration phases/glacial period.

### 5.3. Hominin species admixture

The archaeologically distinct Neanderthals of Lesser Caucasus could be routed into the southern European refugium, who through Anatolia reached the Iranian Plateau and penetrated northwards until the Lesser Caucasus. They might be the population who took the back-and-forth movements to the southern latitude, admix with the populations there and generated the new facies of Mousterian namely Zagros- and Levantine Mousterian. In the lack of any fossil and genetic studies, this hypothesis stays unresolved and awaits more evidence. The location of SCC between Caucasus and Central Asia, gives rise to the hypothesis of SCC as one of the hot spots for tracking hominin admixture and introgression, as it was the case for some faunal species [34, 69]. However, at the present state of knowledge, it is too soon to go further than a hypothesis. Hominin fossils (Neanderthals, *homo sapiens* and Denisovans, or *ghost lineage*) are crucial to encrypt the puzzling picture of hominin settlements in this odd refugium. Conducting intensive archaeological research in this unknown region of south-western Asia is important for our understanding of Neanderthals’ dispersals and admixture with *homo sapiens* and other hominin species. For now, we can hypothesise that interbreeding between the immigrant Neanderthals and the indigenous hominin of SCC was highly possible. SCC was the home range overlapped for Neanderthals from west, *homo sapiens* newcomers from inner parts of the Iranian Plateau via north-south corridors connecting southern and northern slopes and foothills of the Alborz Mountains and other hominins (i.e. Denisovans) from east. However, our knowledge on the inhabitants of the SCC during Pleistocene comes only from the cultural materials (i.e. lithics). Given the assumption of Late Pleistocene population expansion into east through SCC, this area might have been highly populated at the warm and moist stages of MIS 5 [50] and later as refugium during MIS 4 [37, 38, 115] when between 50 and 45 ka the Ust’-Ishim man lived in western Siberia [116]. The genomic history of the Ust’-Ishim man shows that the admixture between the ancestors of the Ust’-Ishim and Neanderthals occurred between ca. 50 to 60 ka [116]. Recent findings from Eskouldar Rockshelter at southern piedmonts of Alborz with the Initial Upper Palaeolithic industry changes our view and show the complex story of human evolution [117, 118].

## 6. Conclusion

Recent research on western Eurasia has increased our knowledge on the migration and dispersal routes of the Neanderthals, their admixture with other hominin types namely Denisovans and *homo sapiens* and colonisation of new ecological niches. The geographical expansion range of human populations largely depends on the spatial distribution of suitable habitats and corridors connecting these habitats. Regarding Neanderthals’ distribution from Europe eastwards, the immediate areas are the piedmonts of the Caucasus and Alborz mountains. However, the role of these corridors for early expansion processes is closely related to the complex pattern of favourable climatic circumstances. Despite highly fluctuated sea level, the SCC remained one of the most important areas for MP population dynamics. During MIS 4 the Caspian Sea level was at the minimum level, large plains have been exposed and provided a large dispersal corridor in the SCC. Moreover, in contrast to the inner basins of the Iranian Plateau, the SCC provided a rich source of freshwater from numerous permanent rivers and terrestrial and marine food resources throughout the year. Together with numerous caves and rockshelters located higher than the boundary of sea level fluctuations, this area was a suitable reservoir and refugium during climatic deterioration. This natural circumstance becomes highly important in terms of the possible interaction between different hominin species arriving from west and east ending up in the meeting point of SCC.

In this regard, our computer-based model identified two major possible routes for the Neanderthals from the gate of Europe in Caucasus to the east. As the map of presence and dispersals of the Neanderthals towards east is at the beginning to be completed, these suggested routes are bounded by major questions and building the future research.

The suggested routes are not only limited for MIS 4, but also conceivable and practical for periods before and after it (i.e. MIS 5–3). We argue that these routes could be repeatedly used by Neanderthals and other hominin species during different phases. In glacial periods these routes lost the connection with each other which resulted into the development of distinct cultural and genetic groups.

We mainly focused on the southern dispersal routes via SCC and hypothesise that during glacial periods, this area due to its exceptional physiogeographic condition could be used as both biogeographical corridor of expansion and settlement. Where other parts of the Iranian Plateau were influenced by cold and dry climatic conditions during MIS 4, SCC benefited from a special condition which made it a remarkable refugium. SCC opens windows of potential contact, Neanderthal demographic influx from western Iranian Plateau and Caucasus into SCC and possible competition with other hominins there. Survey in the SCC as well as the excavation at promising sites, together with detailed climatic reconstruction are needed to confirm that this area has been highly populated at the warm and moist stages of MIS 5 and later as refugium during glacial periods.

The location of Azokh and Teshik-Tash caves at both extremities of the SCC together with the newly discovered sites in this corridor indicate that still promising places in Asia remained largely unexplored, and previously identified sites and materials are in need of renewing studies. Our model shows the complex nature of MP population dynamics in this part of Eurasia than simply characterizing this area was occupied during MP period.
