# Correction: Modelling Neanderthals’ dispersal routes from Caucasus towards east

**DOI:** 10.1371/journal.pone.0303722

**Published:** 2024-05-09

**Authors:** Elham Ghasidian, Anooshe Kafash, Martin Kehl, Masoud Yousefi, Saman Heydari-Guran

There are errors in author affiliations. The correct affiliations are as follows.

Elham Ghasidian^1,2,3^, Anooshe Kafash^4^, Martin Kehl^5^, Masoud Yousefi^4^, Saman Heydari-Guran^1,2,3^

**1** Neanderthal Museum, Mettmann, Germany, **2** DiyarMehr Institute for Palaeolithic Research, Kermanshah, Iran, **3** Institute for Prehistoric Archaeology, University of Cologne, Koln, Germany, **4** Department of Environmental Science, Faculty of Natural Resources, University of Tehran, Tehran, Iran, **5** Institute of Geography, University of Cologne, Cologne, Germany
